# Airway delivery of both a BCG prime and adenoviral boost drives CD4 and CD8 T cells into the lung tissue parenchyma

**DOI:** 10.1038/s41598-020-75734-x

**Published:** 2020-10-30

**Authors:** Daryan A. Kaveh, M. Carmen Garcia-Pelayo, Naomi C. Bull, Pedro J. Sanchez-Cordon, John Spiropoulos, Philip J. Hogarth

**Affiliations:** 1 Vaccine Immunology Team, Department of Bacteriology, Animal & Plant Health Agency (APHA), Addlestone, Surrey UK; 2 Department of Pathology, APHA, Addlestone, Surrey UK; 3 Present Address: Royal Veterinary College, Royal College Street, London, UK

**Keywords:** Immunology, Diseases, Medical research

## Abstract

Heterologous BCG prime-boost regimens represent a promising strategy for an urgently required improved tuberculosis vaccine. Identifying the mechanisms which underpin the enhanced protection induced by such strategies is one key aim which would significantly accelerate rational vaccine development. Experimentally, airway vaccination induces greater efficacy than parenteral delivery; in both conventional vaccination and heterologous boosting of parenteral BCG immunisation. However, the effect of delivering both the component prime and boost immunisations via the airway is not well known. Here we investigate delivery of both the BCG prime and adenovirus boost vaccination via the airway in a murine model, and demonstrate this approach may be able to improve the protective outcome over parenteral prime/airway boost. Intravascular staining of T cells in the lung revealed that the airway prime regimen induced more antigen-specific multifunctional CD4 and CD8 T cells to the lung parenchyma prior to challenge and indicated the route of both prime and boost to be critical to the location of induced resident T cells in the lung. Further, in the absence of a defined phenotype of vaccine-induced protection to tuberculosis; the magnitude and phenotype of vaccine-specific T cells in the parenchyma of the lung may provide insights into potential correlates of immunity.

## Introduction

Tuberculosis (TB) currently claims an estimated 1.5 million lives each year and is the leading cause of mortality by an infectious disease, overtaking HIV in 2015^1^. *Mycobacterium bovis* bacille Calmette-Guérin (BCG) remains the only available vaccine against TB and whilst it provides valuable protection against severe childhood forms of TB^2,3^; meta-analyses of multiple clinical trials indicate widely variable efficacy ranging from 0 to 80%^4^. These data and the continued high burden of TB leads to inevitable questioning of its use, despite being the most widely used human vaccine globally. Consequently, there is a substantial ongoing research effort to identify improved TB vaccines. Nonetheless, due to these aforementioned benefits and recent evidence there may be non-specific protection against other infections^5^; it is unlikely to be withdrawn in the near future, and an improved TB vaccine strategy will likely include BCG^6^. However, after a century of use, we still understand little of the mechanisms which underpin BCG-induced protective immunity. Such knowledge could identify a robust correlate of immunity against TB, still urgently required to help facilitate the rational design of an improved vaccine.

Promising strategies for improved vaccines utilising BCG include boosting immunity with heterologous vaccines, with viral vectors used in several leading approaches^7–11^. Delivery of viral-vectored boosts to the airway mucosa, to align with the natural route of *Mycobacterium tuberculosis* (*Mtb*) infection, induces superior protective efficacy compared to parenteral delivery in many reports^9,11,12^. Numerous studies demonstrate that airway delivery of BCG improves protective efficacy over standard parenteral injection^13–19^, but surprisingly, administration of both prime and heterologous boost by the airway has been much less investigated. To date, this approach has only been reported in promising data from a single study using a BCG-Modified Vaccinia virus Ankara (MVA) prime-boost vaccination^12^.

Intravascular staining (IVS) to discriminate vascular associated cells in live animals has brought a new dimension to cellular immune analyses, allowing concurrent identification of cell phenotype and function together with location within an organ. This technique has identified lymphocyte populations in numerous non-lymphoid tissues including the lung, and facilitated the discovery of tissue-resident memory T cells^20^; implicated in immunity to infectious diseases at both barrier and non-barrier tissues^21,22^.

IVS has shown CD4 T cells induced by *Mtb* infection within the lung parenchyma provide optimal protection compared to CD4 T cells in lung vasculature^23,24^. Tissue-resident T cells in the lung induced by TB vaccines are not yet well described^25–27^. We have shown that intradermal BCG vaccination induces antigen-specific CD4 T cells in the lung^28^, but few reside in the lung parenchyma^29^.

We hypothesised that delivering the BCG prime immunisation via an airway route, rather than parenterally, could further enhance the protective capacity induced by an airway delivered viral vectored boost. Using a murine model, combining a BCG prime-adenovirus boost vaccine^30^ with IVS, we sought to determine whether direct immunisation of the lung mucosa, enhanced tissue-resident immunity within the lung and thus protective immunity to TB challenge.

Here, we demonstrate that compared to parenteral priming, airway delivered BCG induced higher frequencies of antigen-specific multifunctional CD4 and CD8 T cells residing in the lung parenchyma. These observations provide insights into potential correlates of immunity.

## Results

### Airway BCG priming enhances lung parenchymal CD4 T cell responses to boost vaccination

**Figure 1:**
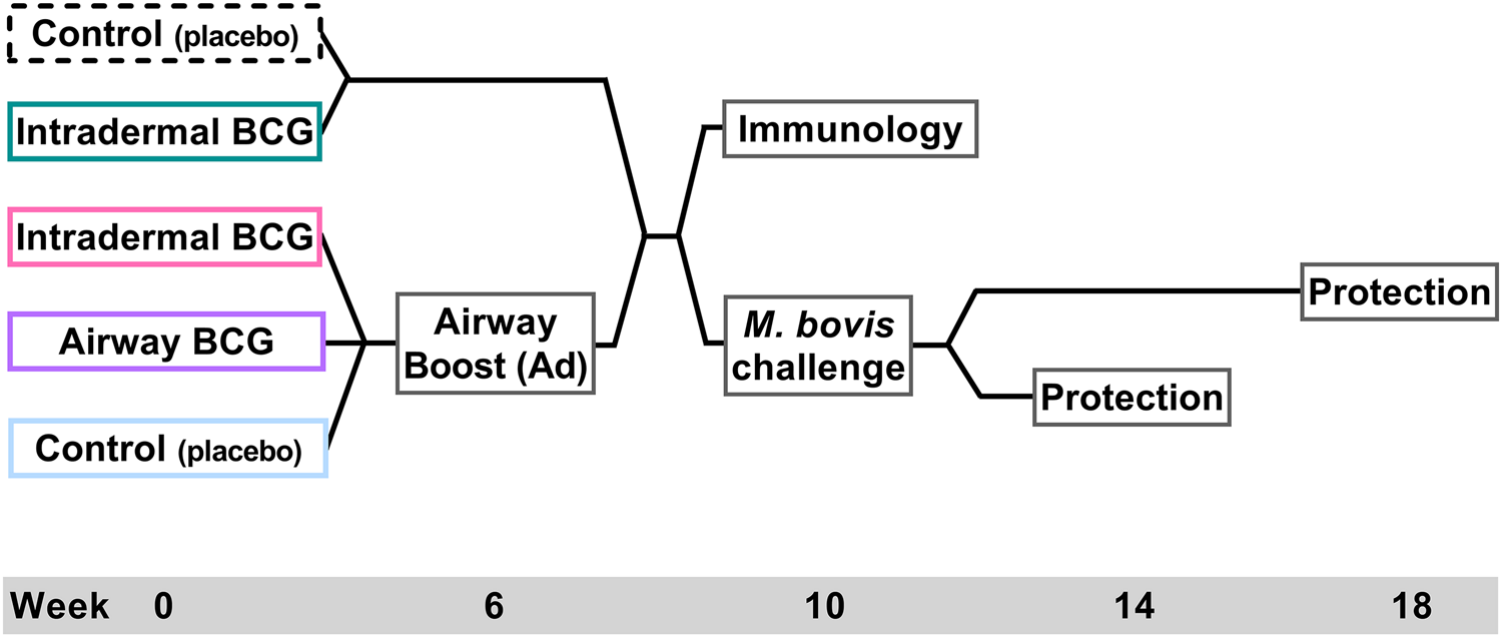
Vaccination regimen schedules. Groups of mice were immunised with a placebo Control or BCG, delivered by either the Intradermal or Airway route. Six weeks later, corresponding groups of mice received a single boost immunisation with adenovirus expressing TB10.4 by the airway route (Ad). Four weeks after the boost for equivalent groups of mice, either the spleen and lung cells were isolated for immunological assessment or mice were challenged with ~ 200 CFU of *M. bovis* by the intranasal route. Bacterial burden in the spleen and lungs of challenged mice was then assessed both 4 and 8 weeks later.

To assess the impact of varying the route of the BCG prime delivery in a BCG prime/boost regimen, (schedule shown in Fig. 1), mice were vaccinated with BCG, by the intradermal or airway (intranasal) route. Six weeks later, they received a single adenovirus boost via the airway. Four weeks after the boost, CD45^+^ vascular cells were stained in vivo; and after isolation and stimulation, antigen-specific vascular and parenchymal spleen and lung cells measured using flow cytometry (gating strategy illustrated in Supplementary Fig. S1 online). Antigen-specific cells (termed cytokine^+^) were identified producing any combination of IFN-γ, IL-2, TNF-α and IL-17.

**Figure 2:**
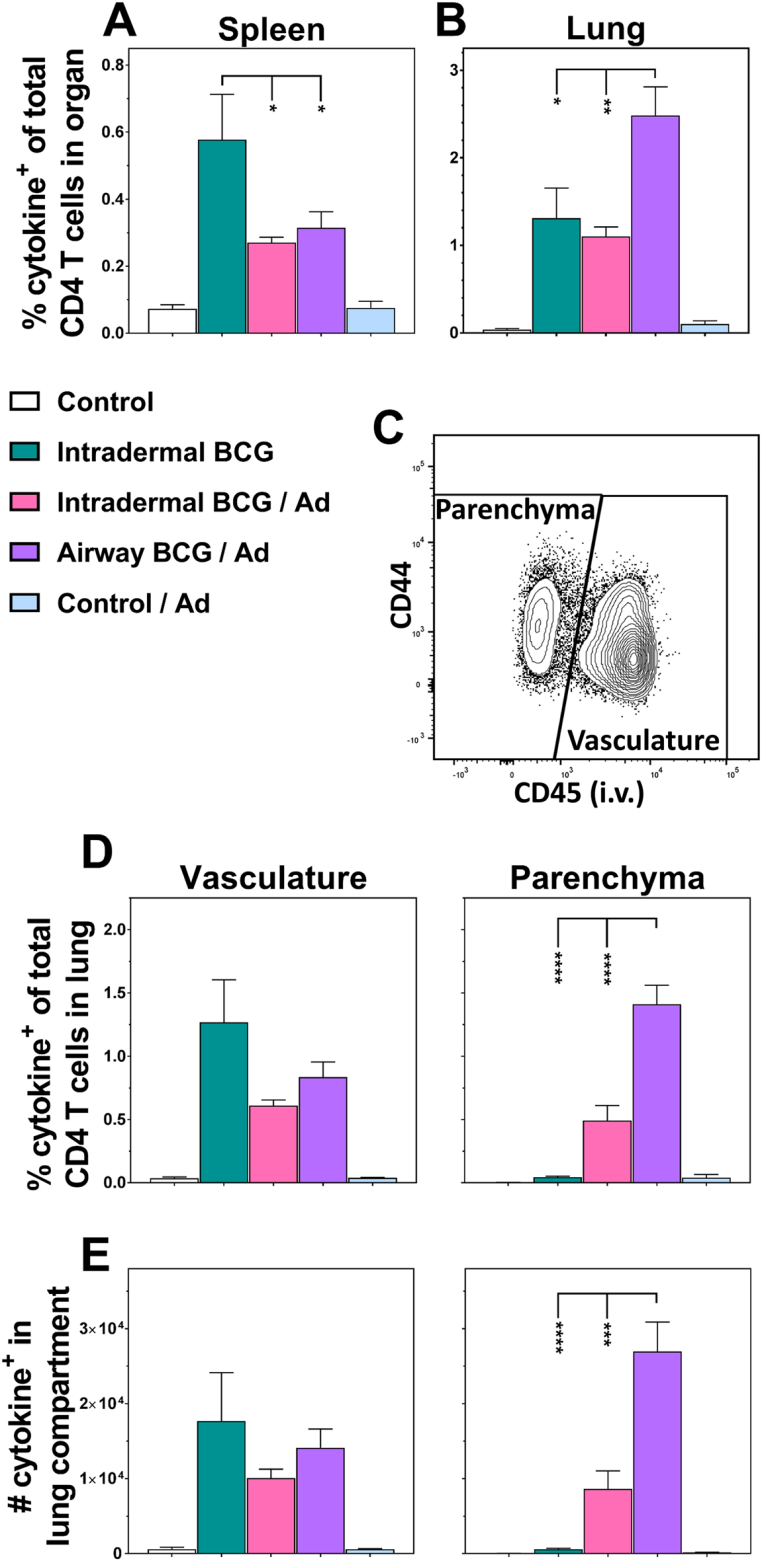
Airway BCG priming enhances lung parenchymal CD4 T cell responses to boost vaccination. Groups of mice were immunised as per Fig. 1 and 4 weeks after the final immunisation they received an i.v. anti-CD45 antibody injection prior to euthanasia. Spleen- and lung-derived cells were in vitro stimulated and interrogated for TB10.4 antigen-specific cytokine production (IFN-γ, IL-2, TNF-α, IL-17) by intracellular staining and flow cytometric analysis (see supplementary Fig. S1). Graphs show the percentage of cytokine^+^ CD4 T cells (exhibiting any combination of IFN-γ, IL-2, TNF-α and IL-17 production) of the total CD4 T cells derived from (**A**) the spleen and (**B**) the whole lung. (**C**) A representative flow cytometry plot illustrating the CD45 intravascular staining obtained by the i.v. injection allowing the differentiation of the whole lung-derived cells into those which resided in the tissue parenchyma (CD45^-^) and those in the lung vasculature (CD45^+^). Graphs showing the (**D**) percentage or (**E**) absolute number of cytokine^+^ CD4 T cells which were resident in the lung vasculature or parenchyma. Bars represent the mean ± SEM (n = 5–11). *p < 0.05, **p < 0.01, ***p < 0.001, ****p < 0.0001, 2-way ANOVA with Tukey’s post-hoc test. Data representative of one experiment.

In the spleen (Fig. 2A), intradermal BCG alone induced a significant frequency of cytokine^+^ antigen-specific CD4 T cells (0.6%, *p* < 0.001 vs. Control). Regardless of the priming route, in animals receiving prime/boost vaccinations these responses were ~ 50% lower (both 0.3%, *p* < 0.05) compared to BCG only.

In the whole lung (Fig. 2B), intradermal BCG prime administration of the prime/boost vaccination did not enhance cytokine^+^ CD4 T cell responses over BCG alone (1.3% vs 1%, *p* < 0.05 vs. Control). In contrast, when the BCG prime immunisation was delivered by airway administration, whole lung cytokine^+^ responses were increased twofold (2.5%; *p* < 0.01 vs. intradermal BCG/Ad).

IVS allowed clear discrimination of vascular/parenchymal lung T cells; vascular-associated cells staining positive for CD45, whilst tissue-resident parenchymal cells remaining unstained^20^ (representative sample Fig. 2C).

Analysis of cytokine^+^ CD4 T cells derived from these compartments of the lung revealed distinct response patterns to varying the route of BCG prime vaccinations (Fig. 2D). Lung vascular cell responses resembled the spleen; intradermal BCG alone induced greater cytokine^+^ cell frequencies than prime/boost (1.3%, *p* < 0.01 vs. Control). In contrast, cells derived from the lung parenchyma displayed a different response pattern; intradermal BCG alone induced negligible cytokine^+^ frequencies (0.04%), increasing tenfold when boosted (0.5%). Most strikingly, varying the BCG prime route to the airway increased the immunogenicity of the boost vaccine 35-fold with parenchymal cytokine^+^ responses representing 1.4% of total CD4 T cells within the lung (*p* < 0.0001 vs. all groups). To confirm these cell frequencies represented real changes in cell populations within the lung, actual cell numbers were calculated, confirming the same response pattern (Fig. 2E).

**Figure 3:**
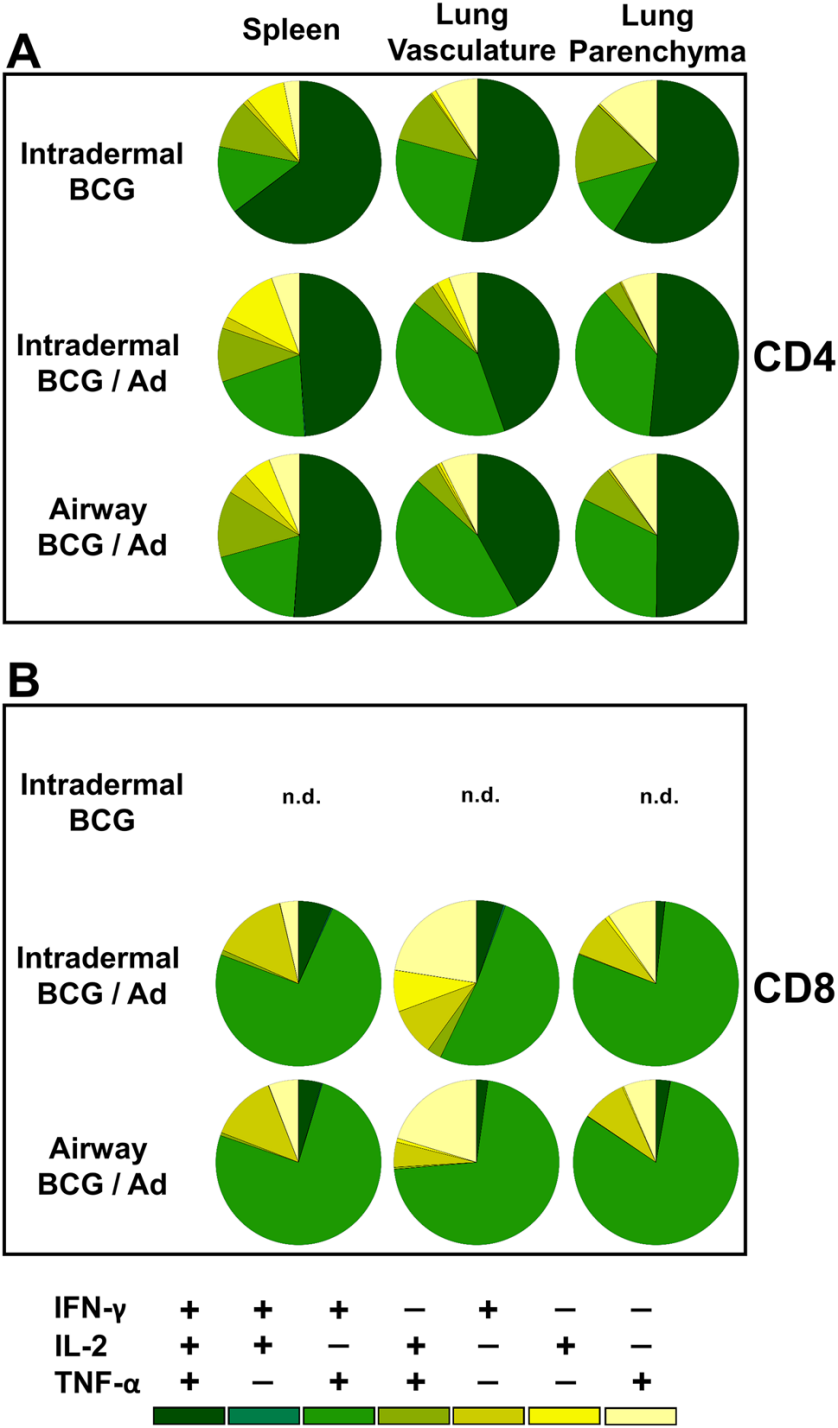
Airway BCG priming of boost vaccination has no marked effect on the proportions of cytokine producing subsets. Cytokine^+^ T cells derived from the spleen, lung vasculature and lung parenchyma, as described in Figs. 2 and 4, were subdivided into subsets producing the different simultaneous combinations of IFN-γ, IL-2 and TNF-α. Pie chart segments represent the mean frequency of the seven individual subsets as a proportion of the total cytokine^+^ population of (**A**) CD4 and (**B**) CD8 T cells in the different tissue compartments (n = 5–11). Only the three BCG vaccine regimens shown for clarity. Data representative of one experiment.

Comprehensive analysis stratified the individual subsets of cytokine^+^ CD4 T cells producing the different simultaneous combinations of IFN-γ, IL-2 and TNF-α (see Supplementary Fig. S2 online). Although analysed, no significant IL-17^+^ cell population was detected (data not shown). These data, summarised in Fig. 3A, indicate significantly predominant multifunctional CD4 T cells of: IFN-γ^+^IL-2^+^TNF-α^+^, IFN-γ^+^TNF-α^+^ or IL-2^+^TNF-α^+^ functional phenotypes. Individual functional subsets did not associate with specific tissue compartments, or immunisation regimens.

### Airway BCG priming enhances both systemic and lung parenchymal CD8 T cell responses to boost vaccination

**Figure 4:**
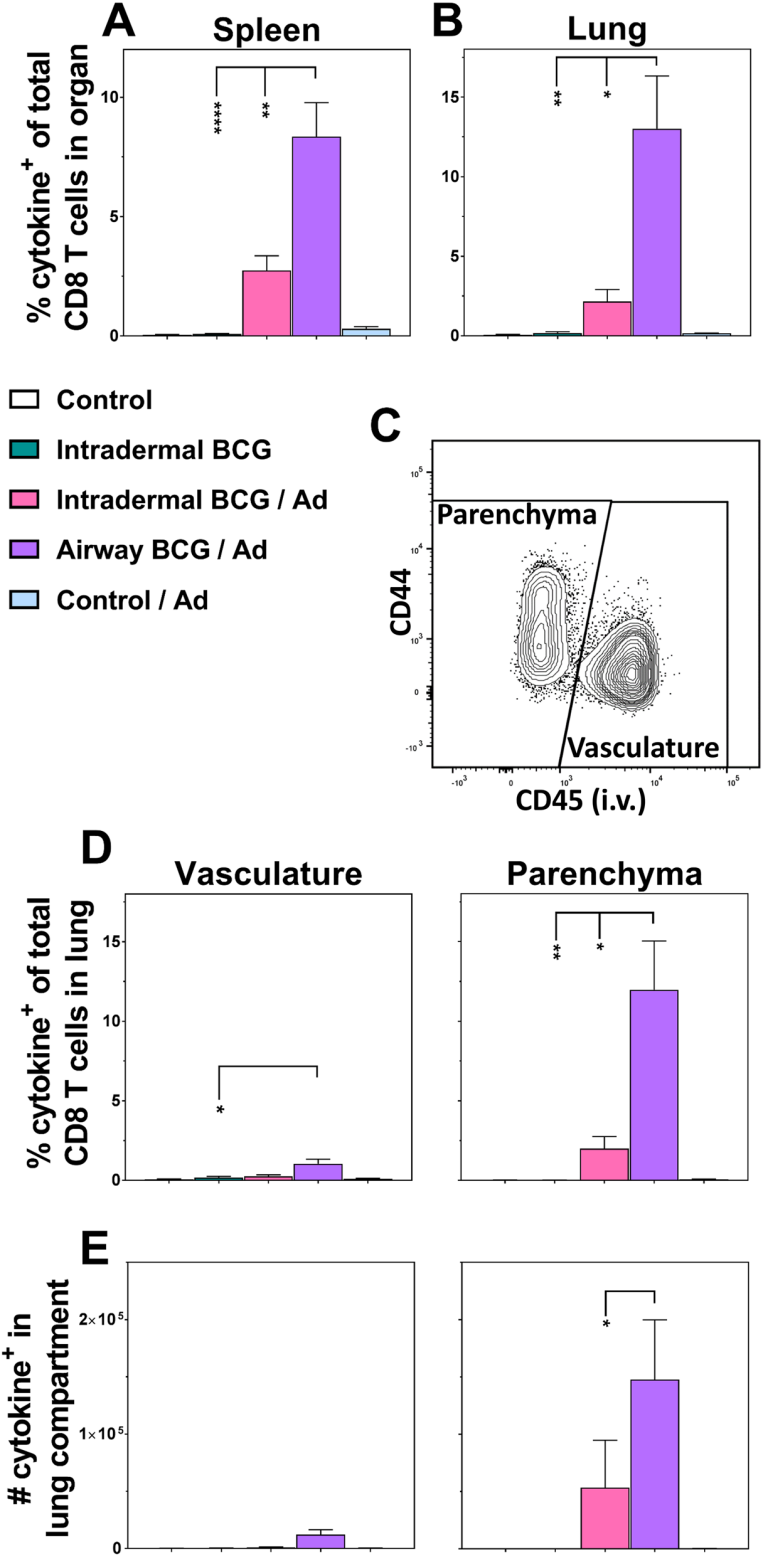
Airway BCG priming enhances both systemic and lung parenchymal CD8 T cell responses to boost vaccination. Groups of mice were immunised as per Fig. 1 and cytokine^+^ cells derived from the spleen and lung detected as described in Fig. 2. Graphs show the percentage of cytokine^+^ CD8 T cells (exhibiting any combination of IFN-γ, IL-2, TNF-α and IL-17 production) of the total CD8 T cells derived from (**A**) the spleen and (**B**) the whole lung. (**C**) A representative flow cytometry plot illustrating the CD45 intravascular staining. Graphs showing the (**D**) percentage or (**E**) absolute number of cytokine^+^ CD8 T cells which were resident in the lung vasculature or parenchyma. Bars represent the mean ± SEM (n = 5–11). *p < 0.05, **p < 0.01, ***p < 0.001 ****p < 0.0001, 2-way ANOVA with Tukey’s post-hoc test. Data representative of one experiment.

Immunisation with intradermal BCG alone failed to induce significant systemic (spleen) antigen-specific cytokine^+^ CD8 T cells, with responses remaining at background levels (0.09% total spleen CD8, Fig. 4A). In contrast, boosting intradermal BCG enhanced systemic CD8 T cell responses 30 fold (2.7% total spleen CD8). However, delivering the BCG prime immunisation by airway; this response was further increased to almost 100 fold (93 fold, 8.4% total spleen CD8, *p* < 0.01).

When viewed as a whole organ, antigen-specific CD8 T cells derived from the lung (Fig. 4B) mirrored that of the spleen. Responses to intradermal BCG alone were minimal (0.18% of lung CD8 T cells), but enhanced tenfold by boosting (2.1% of lung CD8 T cells) and with an order of magnitude increase (72 fold) when BCG prime was delivered via the airway (13% of lung CD8 T cells, *p* < 0.01).

Compartmentalising lung CD8 T cells by IVS staining revealed the background level response induced by BCG alone was lung vasculature derived (0.17% of total lung CD8 T cells; Fig. 4D). In contrast to the spleen, lung vascular cytokine^+^ CD8 T cell responses were only significantly boosted (~ sixfold) when the BCG prime was delivered via the airway (1.7%, *p* < 0.05, Fig. 4D). Analyses of the lung parenchyma-derived cells revealed, that whilst BCG alone failed to induce significant responses (0.01% total lung CD8 T cells), the pattern of responses to both boosting BCG and varying the route of BCG in the prime/boost reflected the response pattern observed in the whole lung. Intradermal BCG prime of the boost induced a 20 fold increase in the parenchymal responses (2% total lung CD8 T cells), while varying the BCG prime route to airway, significantly increased this response 120 fold (12% total lung CD8 T cells, *p* < 0.05). These frequency changes were confirmed by cell numbers (Fig. 4E).

CD8 T cell functional subset stratification (Fig. 3B and Supplementary Fig. S3 online) indicate CD8 T cell responses differed from CD4 T cells with significant populations of bifunctional IFN-γ^+^TNF-α^+^ cells predominating. Functional subsets were not specific to the tissue location, and while only the BCG prime/boost vaccination regimens induced multifunctional CD8 T cells, the route of BCG priming did not affect this stratification.

### Airway BCG priming confers a more sustained protective capacity to the boost vaccination

**Figure 5:**
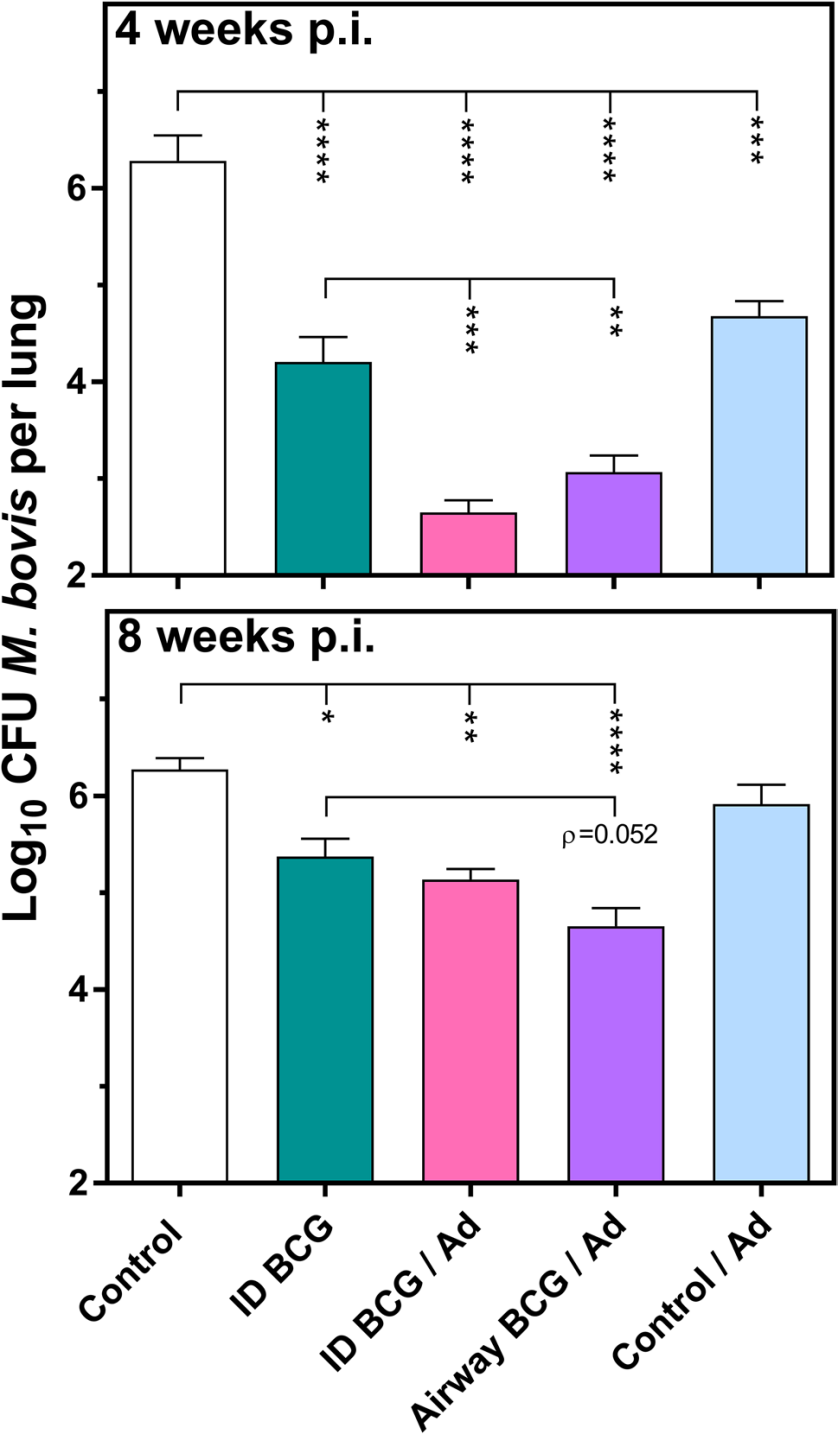
Airway BCG priming confers a more sustained protective capacity to the boost vaccination. Groups of mice were immunised and then challenged with ~ 200 CFU M*. bovis* as per schedule in Fig. 1. Four and eight weeks later, the lungs of individual mice in equivalent groups were removed, homogenised and bacteria enumerated. Bars represent the mean Log_10_ CFU ± SEM (n = 7–14). *p < 0.05, **p < 0.01, ***p < 0.001 ****p < 0.0001, 1-way ANOVA with Tukey’s post-hoc test. Data representative of one experiment.

In order to assess the influence of administering the BCG prime via the airway on protective efficacy, one month after the last vaccination equivalent groups of mice were also challenged with ~ 200 CFU of *M. bovis* by the intranasal route. Bacterial burden in the spleen and lungs was then assessed both at 4 and 8 weeks later. The result for the lungs is illustrated in Fig. 5 and those for the spleen in Supplementary Fig. S4 online.

At 4 weeks post-challenge the *M. bovis* burden in control mice was 6.3 Log_10_ CFU in the lungs and 3.8 Log_10_ CFU in the spleen. Intradermal BCG alone induced protection of 2.1 Log_10_ CFU in the lungs (*p* < 0.0001 vs*.* Control) and 1.5 Log_10_ CFU in the spleen (*p* < 0.05 vs. Control). Intradermal BCG prime administration of the prime/boost vaccination led to an increase in this protection by a further 1.6 Log_10_ in the lungs (*p* < 0.001 vs*.* intradermal BCG) and 0.8 Log_10_ CFU in spleen (not statistically significant vs. intradermal BCG). When the BCG prime immunisation was delivered by airway administration it conveyed equivalent increases in protection over intradermal BCG alone: 1.1 Log_10_ CFU in the lungs (*p* < 0.01 vs. Intradermal BCG) and 1.1 Log_10_ CFU in spleen (*p* < 0.05 vs*.* Intradermal BCG). Of note, the group which received a Control/Ad did exhibit a protective effect approaching that of Intradermal BCG alone: of 1.6 Log_10_ CFU in the lungs (*p* < 0.001 vs*.* Control) and 0.6 Log_10_ CFU in spleen (not statistically significant vs. Control).

At 8 weeks post-challenge, the bacterial burden in control mice remained at 6.3 Log_10_ CFU in the lungs, but had increased to 5.4 Log_10_ CFU in the spleen. The protection afforded by intradermal BCG alone had decreased to 0.9 Log_10_ CFU in the lungs (*p* < 0.05 vs*.* Control) and had remained at 1.5 Log_10_ CFU in the spleen (*p* < 0.001 vs*.* Control). The added protection afforded by both the prime/boost vaccinations had increased to 1.5 Log_10_ CFU in the spleen (*p* < 0.01 vs. Intradermal BCG). However, in the lungs, intradermal administration of the BCG prime no longer offered a protective advantage over intradermal BCG alone (0.2 Log_10_ CFU, *p* = 0.9 vs. intradermal BCG), whilst in contrast using airway administration there remained a discernible reduction in bacterial burden, (0.7 Log_10_ CFU, *p* = 0.0517 vs. intradermal BCG).

### Airway BCG priming of the boost vaccination displays an increased inflammatory reaction to challenge

**Figure 6:**
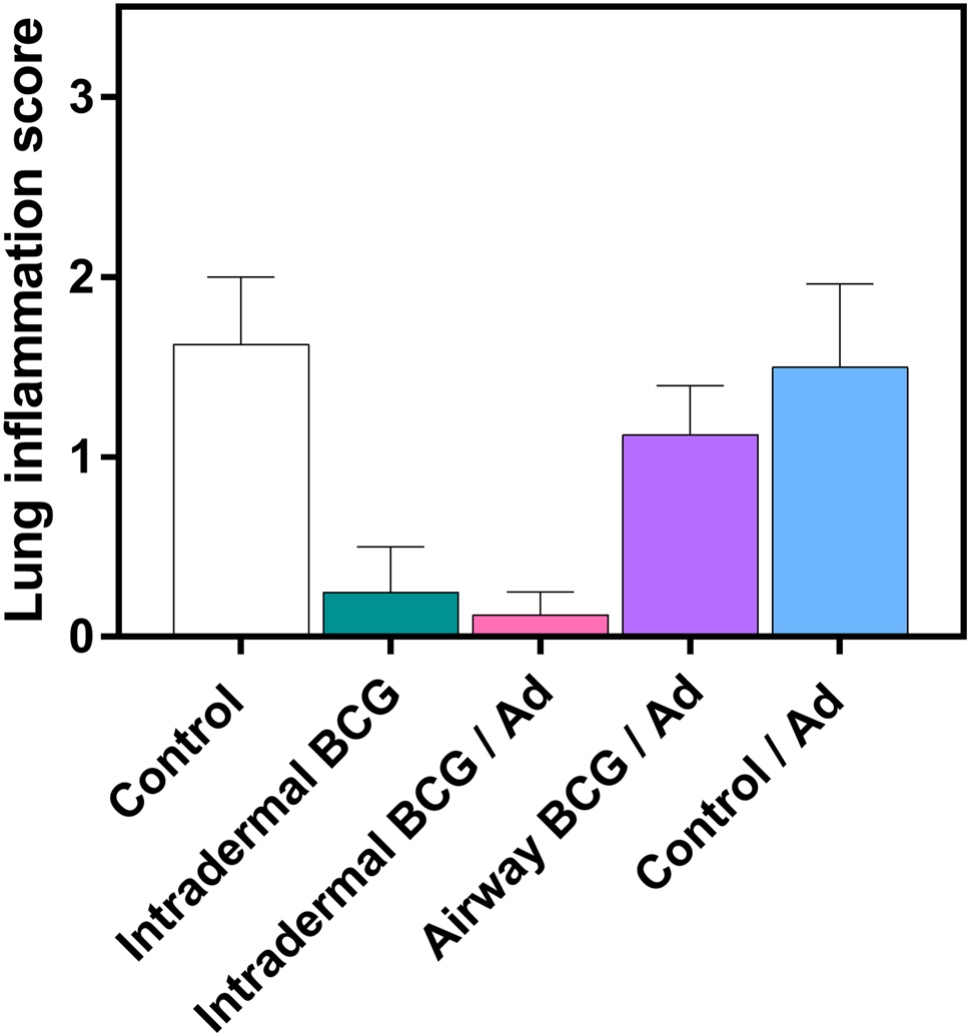
Airway BCG priming of the boost vaccination displays an increased inflammatory reaction to challenge. Groups of mice were immunised and then challenged with *M. bovis* as per Fig. 1, 8 weeks later the right post-caval pulmonary lobes were taken for histopathological examination using H & E staining. Bars represent the mean score of the percentage area of sections from each group exhibiting granulomatous inflammation (n = 8–16). Differences not statistically significant, Kruskal–Wallis test. Data representative of one experiment.

In addition to the assessment of bacterial burden, samples of lung tissue from the same animals were also taken for histopathological analysis to assess the tissue pathology induced by the challenge. Lung tissue sections were assessed for granulomatous inflammation visualised with Haematoxylin and Eosin (H & E) staining. Quantitative analysis of the mean score of the percentage area exhibiting this inflammation revealed no differences between vaccine regimen groups at 4 weeks post-challenge (data not shown). Differences were apparent at 8 weeks post-challenge (Fig. 6), although not statistically significant. The mean pathology score in the lung sections from mice in the Control (1.6) and Control/Ad (1.5) regimens were greater compared to those which received intradermal BCG alone (0.3). Mice which received intradermal BCG prime administration of the boost vaccination had similarly deceased pathology score (0.1), but interestingly, those which were primed by airway administration displayed a mean pathology score closer to that observed in the two control regimens (1.1). In nearly all animals, granulomatous inflammation was accompanied by the presence of mycobacteria observed as acid-fast bacilli (data not shown).

### Increased PD-1 expression associates with cytokine^+^ T cells from the lung parenchyma

**Figure 7:**
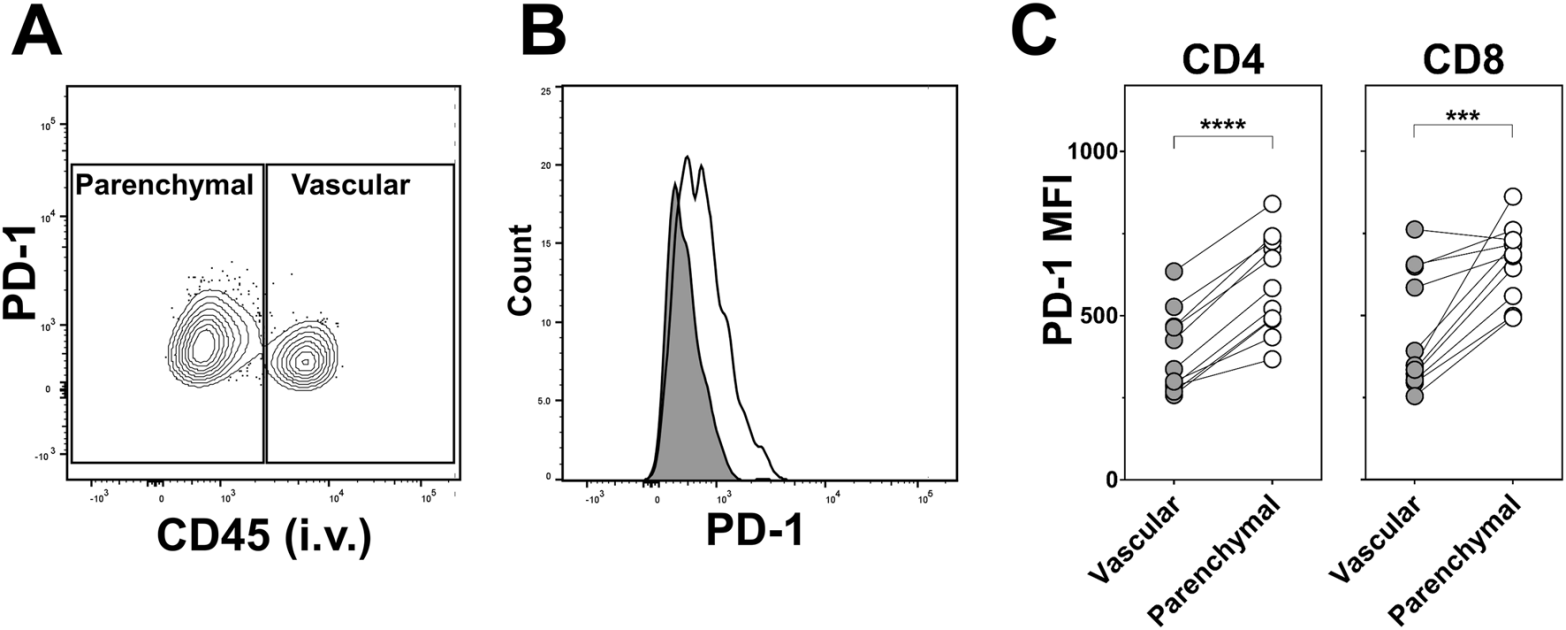
Increased PD-1 expression associates with cytokine^+^ T cells from the lung parenchyma. Groups of mice were immunised as per Fig. 1 and cytokine^+^ CD4 and CD8 T cells derived from the lung parenchyma and lung vasculature compartments detected as described in Figs. 2 and 4. Cells were simultaneously stained for surface expression of PD-1. Representative flow cytometry (**A**) contoured bivariate plot and (**B**) histogram both showing the differential expression of PD-1 by cytokine^+^ CD4 T cells derived from the vascular (CD45^+^, grey filled line) and parenchymal (CD45^-^, unfilled line) compartments of the lungs of mice receiving the airway BCG/Ad regimen. (**C**) Graphs showing the median fluorescence intensity (MFI) of PD-1 staining of cytokine^+^ CD4 and CD8 T cells derived from the two lung compartments of mice receiving this vaccine regimen. Circles represent individual animals (n = 11). ***p < 0.001 ****p < 0.0001, two-tailed paired t-test. Data representative of one experiment.

Defined predictors of protection are not available for TB vaccines, but lung tissue-resident cells may play a role. These responses are not yet well understood, model studies of *Mtb* infection^31^ propose a role for the inhibitory receptor, programmed cell death-1 (PD-1) in protective responses against TB within the lung. To investigate this in the context of enhanced protection and further characterise the antigen-specific parenchymal T cells associated with that protection, expression of PD-1 was examined on the cytokine^+^ CD4 and CD8 T cells derived from the two lung compartments. This was carried out prior to challenge, concurrently with the analysis described for Figs. 2 and 4. There was no detectable association of the level of PD-1 expression by cytokine^+^ cells between the different vaccine regimens and expression of PD-1 was not unique to any specific cytokine producing cell subset (data not shown). Figure 7A illustrates the differential expression of PD-1 by compartmentalised lung cytokine^+^ CD4 T cells derived from mice which received the airway BCG prime. Comparison of the PD-1 staining intensity on these cells subdivided by the two compartments (Fig. 7B,C) revealed that for both cytokine^+^ CD4 and CD8 T cells the median fluorescence intensity (MFI) of PD-1 was on average 1.6 fold (*p* < 0.0001) and 1.7 fold (*p* < 0.001) greater, respectively, on those derived from the lung parenchyma compared to the vasculature, supporting its association with tissue-resident cells.

## Discussion

The need for a new TB vaccine is urgent and leading strategies proceeding into clinical trials are BCG prime—heterologous boost regimens. In experimental studies, mucosal delivery via the airway has demonstrated greater efficacy than parenteral delivery. This is the case when BCG^13–19^ or viral vectors^32–34^ are used alone and also when viral vectors are used to boost a parenteral BCG immunisation^9,11,12^. Here, we investigated the effect of delivering both the BCG prime, and adenoviral boost vaccination via the airway in a murine model of *M. bovis* challenge. Delivery of prime via the airway (intranasal) induced greater migration of antigen-specific multifunctional CD4 and CD8 T cells into the lung parenchyma than the parenteral (intradermal) route, and displayed an indication that it could convey an improved protective outcome.

Despite sustained research in this area, the protective immune response against TB is still not well understood. It is clear that the primary protective immune response requires antigen-specific CD4 T cells producing IFN-γ (reviewed in^35^). This may support a CD8 T cell response, which is required for optimal protection^36^, however, the exact role of CD8 T cells is not well established. Studies of vaccination-induced CD8 T cells report that superior protection using viral vectored boosting associates with CD8 T cell responses^11,30,37^. Therefore, higher frequencies of both antigen-specific CD4 and CD8 T cells appear to associate with better protection. Multifunctional antigen-specific T cells are also potential correlates of vaccine mediated protection^38^ and our previous work using *M. bovis* challenge models support this^28,39^. However, such generalised measurements do not offer a robust correlate of protection^40^ and require increased granularity.

We have previously shown parenteral (intradermal) BCG immunisation induces a persistent population of antigen-specific CD4 T cells in the lungs of mice^28^ and others provide evidence that these cells associated with protection^41^. This has also been shown for CD8 T cells resident in the lung induced by adenoviral vaccination, which appear to proliferate in situ^42^. The location of antigen-specific T cells at the site of the challenge infection has been proposed as a key element in vaccine induced protection against TB, by accelerating initial responses to infection^43^. Horvath et al. report that BCG-induced lung tissue-resident (likely predominantly vascular) CD4 T cells exhibit delayed proliferation, which can be ameliorated by prior residence in the airways, improving protective outcome^44^. This infers that primed antigen-specific T cells located as close as possible to the lung airway may be able to offer an increased protective capacity.

The development of IVS allowed discrimination of tissue-resident cells into those which associate with the vasculature and the parenchyma^20^. Antigen-specific lung parenchymal CD4 T cells are more protective than lung vascular cells in both *Mtb*^23,24^ and respiratory virus infection models^45^. This supports the hypothesis that vaccination which can induce antigen-specific T cells in the lung parenchyma may be more protective to respiratory infection. Protection by a parenteral subunit TB vaccination has associated with parenchymal multifunctional CD4 T cells induced post-challenge^25,26^. Few studies have localised T cells by IVS in the context of TB vaccination prior to challenge. The majority of antigen-specific T cells isolated from whole lung are typically resident in the lung vasculature rather than the parenchyma^46^, as was confirmed for parenteral BCG vaccinates in the current study and a recent publication^29^. This more systemically distributed response, as observed in the spleen, was driven to the lung parenchyma with our adenoviral boost and amplified by airway priming.

Polarisation of antigen-specific responses toward CD8 T cells is a hallmark of virally vectored vaccines. Those induced in the lung lumen by an airway, but not parenteral, adenoviral TB vaccine is associated with its superior protective effect^34,42^. As the frequency of these cells correlates with those in the lung parenchyma^27^, the compartments are likely closely associated. This is supported by the ability of airway delivered BCG to induce antigen-specific CD4 T cells in both the lung tissue parenchyma and the airway lumen^19^.

In the current study, at 4 week post-challenge airway prime/boost did not increase protection over that provided by parenteral prime/boost, although both approaches were significantly better than BCG alone. Evaluation at 8 weeks post-challenge expands the window in which enhanced protection can be observed, as the level of protection induced by parenteral BCG wanes from 4 to 8 weeks post-challenge, as previously reported in *Mtb*^47,48^ and *M. bovis*^49^ challenge models. We observed that a reduction in bacterial burden (better than BCG alone) was more evident in the airway primed group, than the parenteral primed group. Although this was not quite statistically significant (*p* = 0.0517), we believe these data are compelling when viewed alongside the immunological parameters measured. To further support this finding, further experimentation employing more prolonged challenge or survival experiments could provide valuable insight. The equivalent level of protection for both boost regimens in the spleen at 8 weeks post-challenge may indicate parenteral BCG priming protects against bacterial dissemination to a degree which cannot be improved upon by airway BCG priming, observed within the parameters of our model. In previous work, we have observed airway BCG alone is no better than parenteral BCG in the spleen at 4 weeks post-challenge^19^.

In our model we employ challenge infection with *M. bovis.* With its high degree of genetic homology to *Mtb*^50^, a broader host range and greater virulence^51,52^ (and unpublished data), it could provide a useful surrogate for laboratory strains of *Mtb* in murine models. To clarify this, it would be highly informative to directly compare the vaccine-induced protection provided by low dose aerosol challenge of mice with these two mycobacterial species.

Lung histopathology indicated that at 8 weeks post-challenge there was an increased trend for granulomatous inflammation in groups primed via the airway compared to the parenteral route. Although airway delivery of BCG alone is able to directly cause a measurable degree of such lung pathology^53,54^, that observed in this study appeared to be associated only in response to challenge as was not present at 4 weeks post-challenge, when BCG presence in the lung would be expected to be higher. This may indicate enhanced immune responses, or more probable, a localised inflammatory environment induced by BCG bacilli. Further experimentation may clarify if pathology is maintained below that of unprotected control animals. In agreement, others have observed post-*Mtb* challenge, animals receiving airway delivered BCG, displayed increased lung pathology compared to parenteral BCG^54^, highlighting the importance of evaluating vaccine safety in addition to efficacy and immunogenicity.

ICS staining and analyses reveal distinct functional phenotypes for cells which associate with enhanced protection, although it must be stressed, not a direct correlate. Our data demonstrate, BCG vaccination by any route induces antigen-specific multifunctional CD4 T cell (IFN-γ^+^IL-2^+^TNF-α^+^, IFN-γ^+^TNF-α^+^ or IL-2^+^TNF-α^+^) effector/effector memory phenotypes as described previously^28,39^. Antigen-specific CD8 T cells were only induced by regimens including adenovirus vaccination; and were less multifunctional (IFN-γ^+^TNF-α^+^, IFN-γ^+^ and TNF-α^+^), similar to that observed in our previous work^30^, although at a much higher frequency.

We observed no correlation between specific lung location and T cell functional phenotype in any vaccine regimen. In contrast, *Mtb* infection induces specific CD4 T cell populations in the lung parenchyma with fewer IFN-γ producing cells^23^. In contrast to our use of in vitro antigen stimulation, this study utilised MHC Class II tetramers to identify antigen-specific cells which requires the use of direct ex vivo ICS staining and data cannot be directly compared.

We were unable to undertake more comprehensive cell surface phenotyping in combination with ICS and IVS, but did measure expression of the immune checkpoint molecule PD-1 on tissue-resident antigen-specific T cells. Expression of PD-1 was enhanced on lung parenchyma resident cytokine^+^ CD4 and CD8 T cells, compared to vascular associated cells, in agreement with other reports^23,24,31^. PD-1 expression on tissue-resident cells during *Mtb* infection may regulate proliferative ability^55^, and by downregulating IFN-γ induced pathology, protect from lethal disease^24,31^, although its role in vaccination-induced protection in not yet established. The observed lower magnitude in changes to PD-1 expression, as reported elsewhere^56^, may represent differences between infection and vaccination. In an effort to discover both the mechanisms and predictors of vaccine mediated protection, further concurrent analyses of other markers associated with parenchymal T cells, such as CXCR3^23,27^, would also be informative.

The antigen-specific lung parenchymal T cells in our study are likely to represent both effector and/or effector memory T cells^29^, but based solely upon location in the lung parenchyma, we are as yet unable to confirm whether they represent canonical tissue-resident memory T cells (T_RM_)^21^ and merits further investigation in TB vaccine models.

Live BCG bacilli are able to persist in vaccinated mice for at least 18 months in the secondary lymphoid organs with little lung involvement after parenteral BCG immunisation^28^. It is therefore possible BCG administered by the airway may cause similar persistence in the lung tissue^53,54^ and thus influence antigen-specific T cells in the lung parenchyma via localised inflammatory environments^54^. Whether this impacts immune protection merits further investigation in vaccine studies using BCG. It is formally possible that such BCG bacilli within the lungs could lead to underestimation of protective efficacy by adding to the mycobacterial burden. To rule this out, such techniques as genetic sequence analysis of recovered bacilli or use of selective culture media would be worthwhile in future studies.

Adenoviral vaccination alone did provide significant protection at 4 weeks post-challenge, although not to the extent described by others^11,47,57^. There are several potential reasons for this: differences in antigens (Ag85a vs. TB10.4); vaccine preparation; or the challenge (*M. bovis* vs. *Mtb*). This protection was not associated with substantial antigen-specific T cell responses, however the small frequency of CD4 T cells in the lung parenchyma and CD8 T cells in the spleen may play a role.

It should be noted, our study was not designed to identify superior vaccine candidates, but to evaluate the potential of an airway prime—airway boost vaccination regimen and investigate immune mechanisms in a ‘better than BCG’ scenario. Our data demonstrate that this approach could offer an advantage over parenteral BCG prime due to the induction of increased frequencies of antigen-specific CD4/8 T cells located specifically in the lung parenchyma, as such cells have been identified to have an increased protective capacity against *Mtb* and could play a significant role. Therefore, identifying lung compartment location of highly focussed T cell responses (e.g. multifunctional CD4/8 T cells with downregulated inhibitory functions) is a promising approach. It identifies a strategy whereby combining: the specific location of vaccination-induced T cells; surface and functional phenotype mapping; and ‘better than BCG alone’ vaccination regimens which increase the frequency of such cells in the lung parenchyma, may identify key responses to further improve rational vaccine design against TB.

## Materials and methods

### Ethics

All animal work was carried out in accordance with the UK Animal (Scientific Procedures) Act 1986; under appropriate licences. The study protocol was approved by the APHA Animal Use Ethics Committee (UK PCD number 70/6905).

### Animals

Female BALB/c mice were obtained from SPF facilities at Charles River UK Ltd. and used at 8 weeks of age. All animals were housed in appropriate Advisory Committee on Dangerous Pathogens (ACDP) Containment Level 3 (equivalent to BSL3) facilities at APHA, according to the Code of Practice for the Housing and Care of Animals Bred, Supplied or Used for Scientific Purposes^58^. All animals were randomly assigned to treatment groups in appropriate numbers and housed and cared for, including during the challenge period, as previously described^30^.

### Mycobacteria, mycobacterial enumeration, viral vectors and antigens

The vaccination strain was the human vaccine *M. bovis* BCG Danish 1331, reconstituted as per manufacturer’s instructions (SSI, Copenhagen, Denmark) in Sauton diluent (SSI) supplied with the original vaccine. Expression of TB10.4 protein in a type 5 Ad was undertaken as previously described^30^ and prepared for immunisation in PBS.

*M. bovis* strain AF2122/97 was used for all challenge experiments as described previously^39^.

Two immunodominant peptides (Pepscan, Lelystad, The Netherlands), [SSTHEANTMAMMARDT] and [AGYAGTLQSLGAEIAV] of the TB10.4 protein were used for in vitro antigen stimulations as these were previously found to contain the only CD4 and CD8 epitopes induced in this model, respectively^30^.

Mycobacteria were enumerated in aseptically removed spleen and lungs from animals after euthanasia. Organs were homogenised, serially diluted and plated out onto modified Middlebrook 7H11 agar medium as previously described^59^, except that homogenisation was carried out in 1 ml volumes utilising a Precellys 24 and CK28-R tubes (Bertin Instruments, Montigny-le-Bretonneux, France). Bacterial colonies were enumerated 4 weeks later following incubation at 37 °C.

### Immunisation and challenge

There were five separate treatment groups of mice: Control (placebo); Intradermal BCG; Intradermal BCG/Ad; Airway BCG/Ad; and Control/Ad. Groups of mice were immunised with a single injection (50 µl) containing 2 × 10^5^ CFU of Intradermal BCG in the base of the tail, or the same dose of BCG (or diluent only placebo) in 25 µl intranasally (i.n.). We refer to this i.n. delivery as airway, as our previous data demonstrated that 75% of inoculum delivered to the nares entered the lungs^49^. Six weeks later corresponding groups of Control (placebo) or BCG immunised mice were boosted once, with 5 × 10^7^ PFU (25 µl) of Ad-TB10.4 delivered i.n.. Four weeks later, six mice per group were euthanised for immunological analyses, and all remaining mice were challenged with ~ 200 CFU M*. bovis* i.n. as previously described^49^. At both 4 and 8 weeks post-challenge, the bacterial loads in the spleen and lungs of equivalent groups of mice were enumerated as previously described above. The right lung post-caval lobe was first separated for histopathological analysis. All i.n. inoculations were conducted under brief general anaesthesia with Isofluorane.

### Cell isolation and stimulation

Immediately prior to euthanisation by cervical dislocation, 0.075 µg of anti-CD45 – PE (clone: 30-F11, BioLegend, London, UK) was delivered intravenously (i.v.) in 100 µl for exclusive in vivo staining of intravascular cells. Following euthanasia, spleens and lungs were aseptically removed and cells prepared as previously described^39^, except in order to prevent ex vivo staining, the lungs were immediately homogenised with a GentleMACS dissociator (Miltenyi Biotec, Bisley, UK) and the homogenate diluted to 20 ml. Following washing (300 g/8min), all cells were re-suspended at 1 × 10^7^/ml for assays. Cells were cultured with a pool of both TB10.4 peptide antigens (see above) each at a final concentration of 2 µg/ml for all assays in addition to 1ug/ml anti-CD28 (clone: 37.51, BD Biosciences, Oxford, UK).

### Flow cytometry

For intracellular staining (ICS), cells were cultured with the above antigen stimulation as previously described^39^. Subsequently, they were surface stained with pre-titrated antibodies: CD4-APC-H7 (clone: GK1.5, BD Biosciences); CD90.2—efluor450 (clone: 53-2.1) ; CD19—Biotin (clone: eBio1D3); CD16/32—Biotin (clone: 93, Life Technologies, Paisley, UK); PD-1—FITC (clone: 29F.1A12) ; CD8—Alexa Fluor 700 (clone: 53-6.7), CD44—Brilliant Violet (BV) 785 (clone: IM7) and Zombie Aqua Fixable Viability Dye (‘Zaqua’); followed by Streptavidin—BD Horizon BV711 (BD Biosciences). Subsequently, cells were washed, fixed/permeabilised and stained by ICS with IFN-γ—PE-Cy7 (clone: XMG1.2); IL-2—APC (clone: JES6-5H4); IL-17a—BV605 (clone: TC11-18H10.1); and TNF-α—PerCP-Cy5.5 (clone: MP6-XT22, BD Biosciences) as previously described^39^. All antibody conjugates/dyes were purchased from Biolegend except where stated.

Cells were analysed immediately after final staining. Data were acquired using a SORP LSR Fortessa (BD Bioscience) (utilizing a 532 nm laser for PE and PE-Cy7) and analyzed on Flowjo v.10.1 (BD Bioscience) software. All analyses were gated on a minimum of 100,000 live lymphocytes. Compensation was performed using UltraComp eBeads (Life Technologies) according to the manufacturer’s instructions. Fluorescence minus one (FMO) controls were used to set gates for cytokine analyses.

### Histopathological analysis

Lung tissue samples were fixed in 4% buffered formalin solution, routinely processed and embedded in paraffin wax for histopathological studies. Paraffin-embedded tissue blocks were sectioned at 4 μm thickness and stained with haematoxylin and eosin (H & E). A blind histopathologic evaluation was carried out on H & E stained sections by a qualified pathologist. Manual scoring based on the approximate percentage of lung area affected by granulomatous inflammation was carried out as follows: 0% (0); < 5% (1); 5–20% (2); 21–40% (3); 41–60% (4); 61–80% (5); > 80% (6). Serial sections were also stained by the conventional Ziehl–Neelsen staining method in order to confirm the presence of acid-fast bacilli (AFB) in the pulmonary granulomatous lesions.

### Statistical analysis

Bacterial burden data were analysed by 1-way ANOVA and ICS by 2-way ANOVA, all with Tukey’s *post-hoc* test. Prior to analysis, mycobacterial counts were log_10_ transformed (Y = log[Y]) and expressed as log_10_/organ. PD-1 expression was analysed using a two-tailed paired t-test. Histopathological data were analysed by Kruskal–Wallis test. Statistical analyses were carried out using GraphPad Prism 7 software (GraphPad, USA). Differences with a *p* < 0.05 were considered significant and denoted with *, *p* < 0.01 with **, *p* < 0.001 with *** and *p* < 0.0001 with ****.

## Supplementary information

Supplementary Figures.

## Data Availability

The datasets generated during and/or analysed during the current study are available from the corresponding author on reasonable request.
