# Supplementary material for: Airway delivery of both a BCG prime and adenoviral boost drives CD4 and CD8 T cells into the lung tissue parenchyma
Source: Sci Rep. 2020 Oct 30;10:18703. doi: 10.1038/s41598-020-75734-x (PMC7603338; doi:10.1038/s41598-020-75734-x)
Supplement: Supplementary file 1 — Supplementary Figures. [file 41598_2020_75734_MOESM1_ESM.pdf]

Airway delivery of both a BCG prime and adenoviral boost drives CD4 and CD8 T cells into the lung tissue parenchyma

Daryan A. Kaveh, M. Carmen Garcia-Pelayo, Naomi C. Bull, Pedro J. Sanchez-Cordon, John Spiropoulos & Philip J. Hogarth

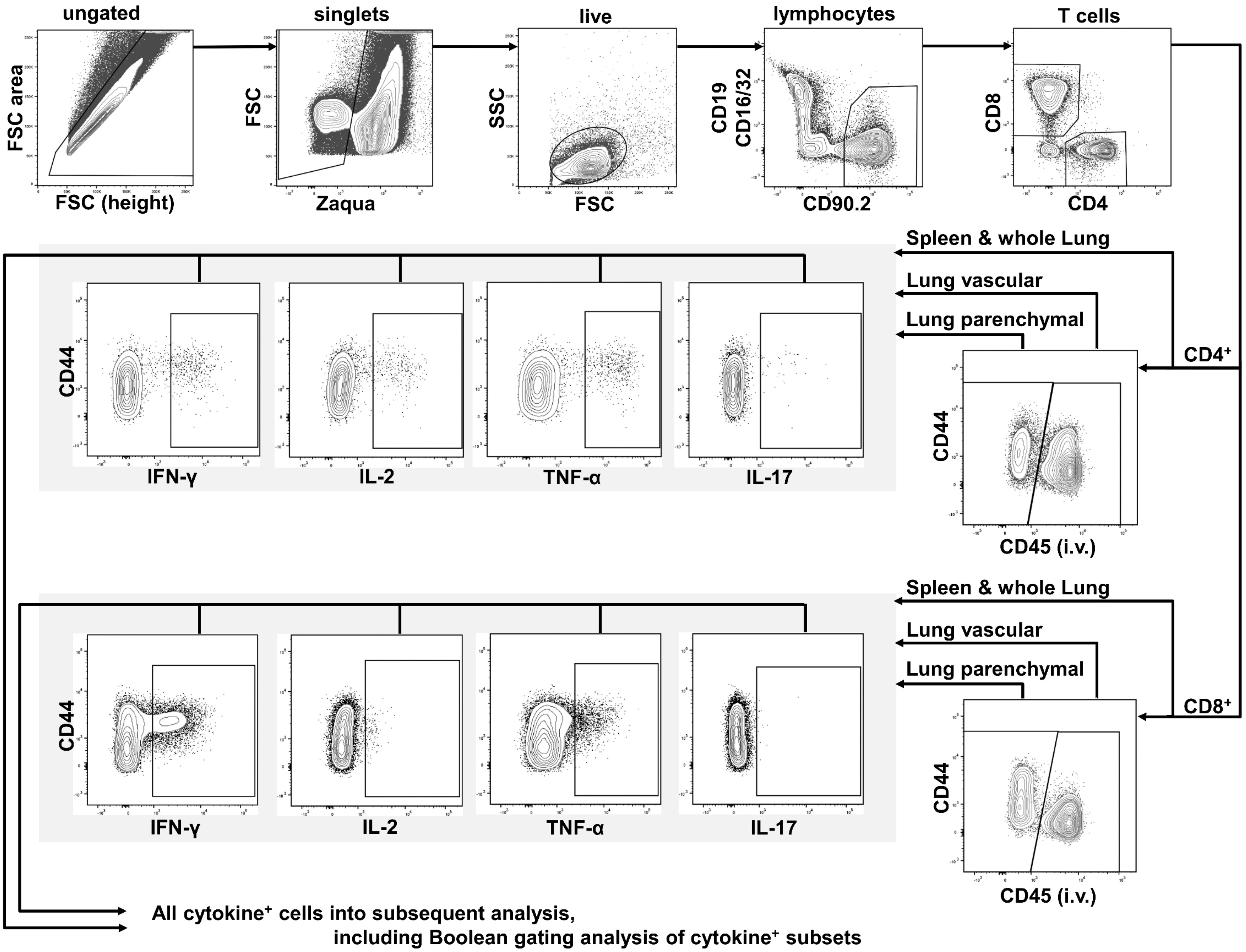

Supplementary Figure S1. Flow cytometry gating strategy. Gating strategy used for identification of CD4 and CD8 T cells producing IFN-γ, TNF-α, IL-2, and IL-17 alone or in any combination. Cells were gated on singlets followed by live cells and then lymphocytes. T cells were identified as CD90.2$^+$ CD19$^-$ CD16/32$^-$ before gating on CD4$^+$ or CD8$^+$ cells. Where lung-derived CD4 or CD8 T cells were additionally subdivided into those residing in the parenchyma or vasculature, CD45$^-$ and CD45$^+$ (intravascular stained) cells were gated, respectively. The use of a bivariate plot vs. CD44 provided increased definition of this and subsequent gating. Antigen-specific CD4 or CD8 T cells were then identified by their production of IFN-γ, TNF-α, IL-2 or IL-17. Boolean gating was then used to identify all cells producing any combination of one or more of these cytokines (termed cytokine$^+$) as well as the individual cytokine$^+$ CD4 and CD8 T cell subsets defined by the different simultaneous combinations.

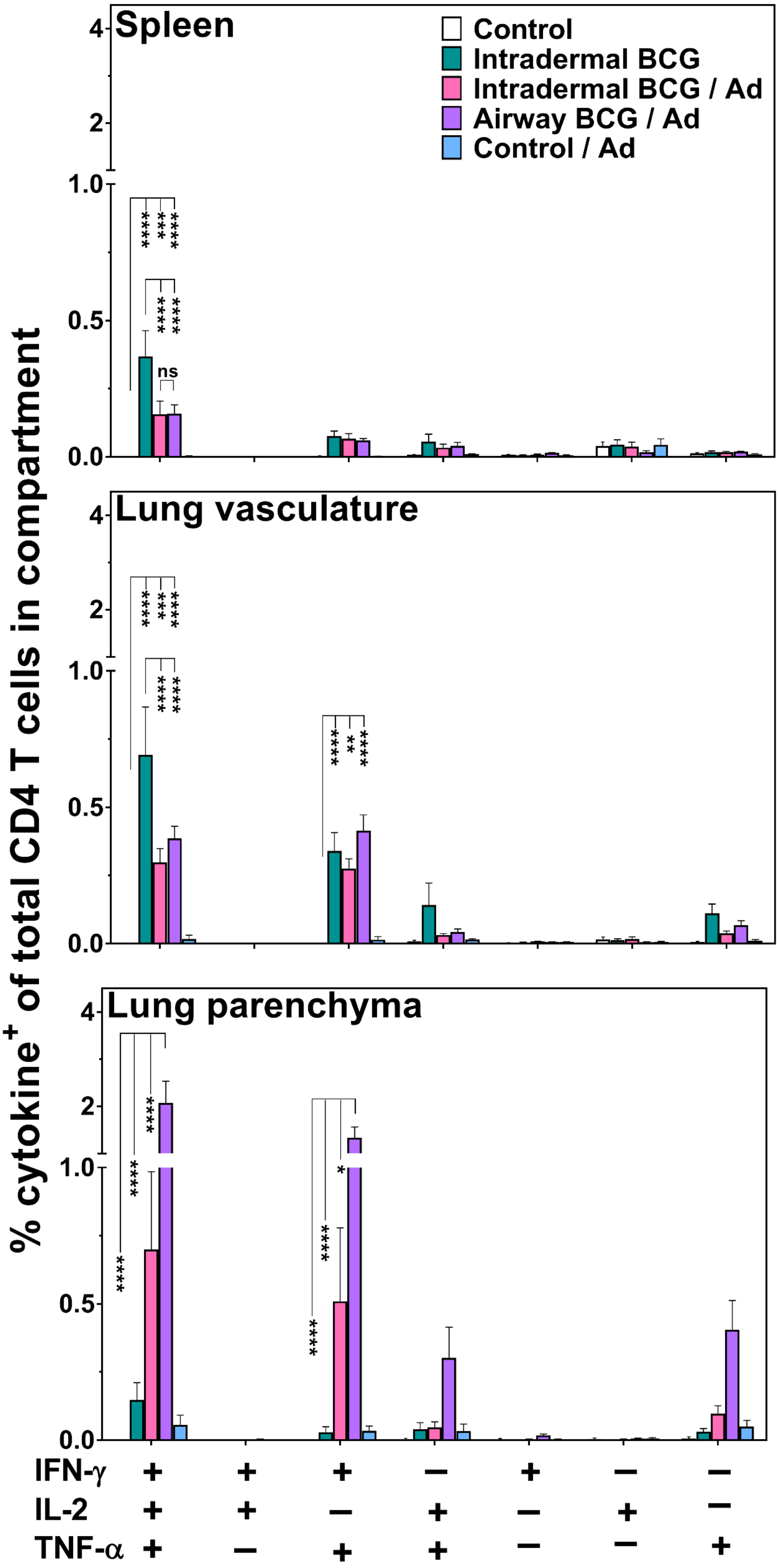

Supplementary Figure S2. Frequency of individual functional subsets of cytokine producing CD4 T cells. Cytokine+ CD4 T cells derived from the spleen, lung vasculature and lung parenchyma were identified as per Fig. 2 and then subdivided into the seven possible individual subsets defined by the different simultaneous combinations of IFN-γ, IL-2 and TNF-α production. Data represent the frequency of each cytokine producing subset as a percentage of the all CD4 T cells in the organ/compartment. Bars represent the mean ±SEM (n = 5-11). * p <0.05, ** p <0.01, *** p <0.001 **** p <0.0001, 2-way ANOVA with Tukey's post-hoc test.

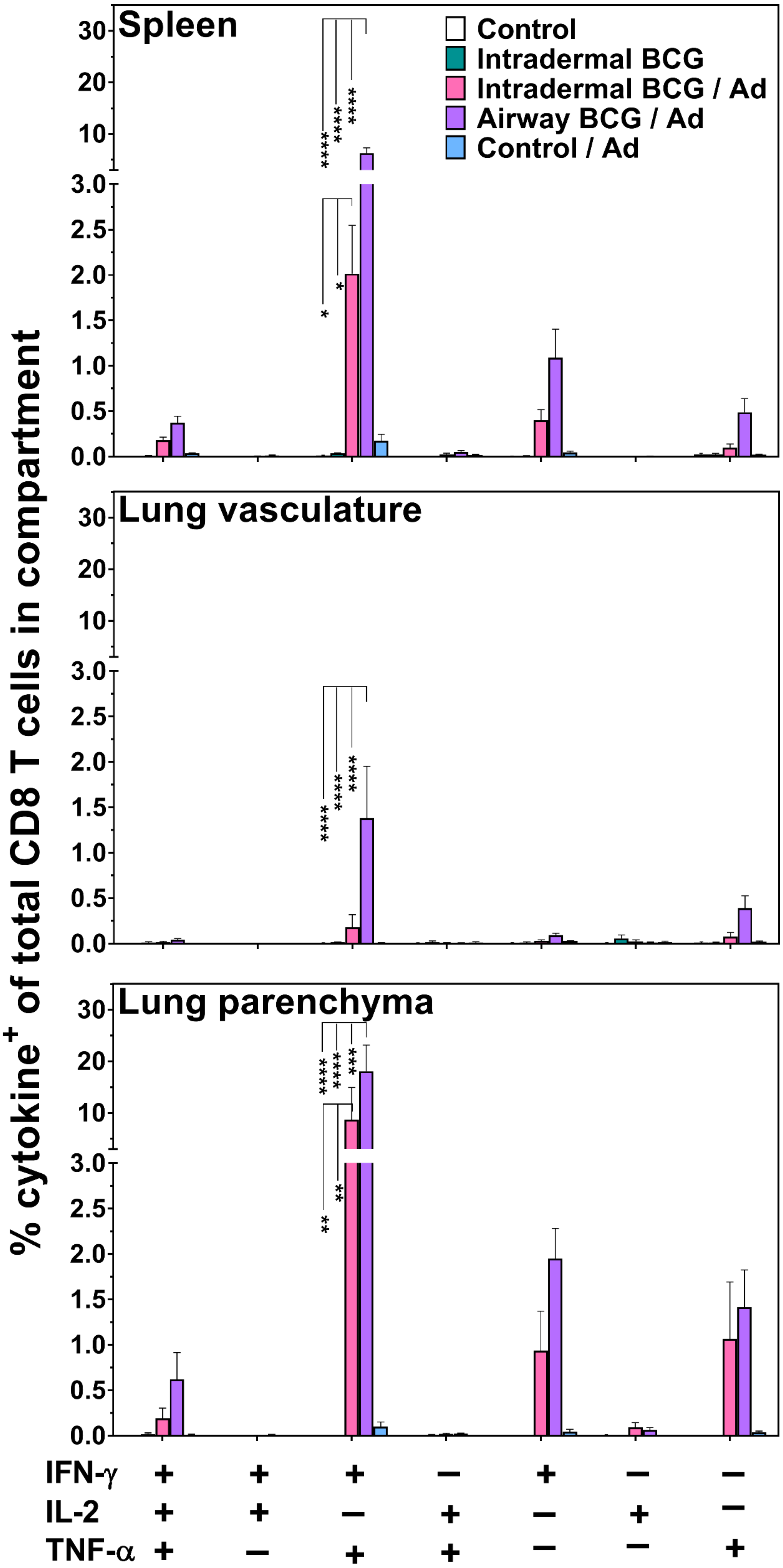

Supplementary Figure S3. Frequency of individual functional subsets of cytokine producing CD8 T cells. Cytokine+ CD8 T cells derived from the spleen, lung vasculature and lung parenchyma were identified as per Fig. 4 and then subdivided into the seven possible individual subsets defined by the different simultaneous combinations of IFN-γ, IL-2 and TNF-α production. Data represent the frequency of each cytokine producing subset as a percentage of the all CD4 T cells in the organ/compartment. Bars represent the mean ±SEM (n = 5-11). * $p < 0.05$, ** $p < 0.01$, *** $p < 0.001$ **** $p < 0.0001$, 2-way ANOVA with Tukey's post-hoc test.

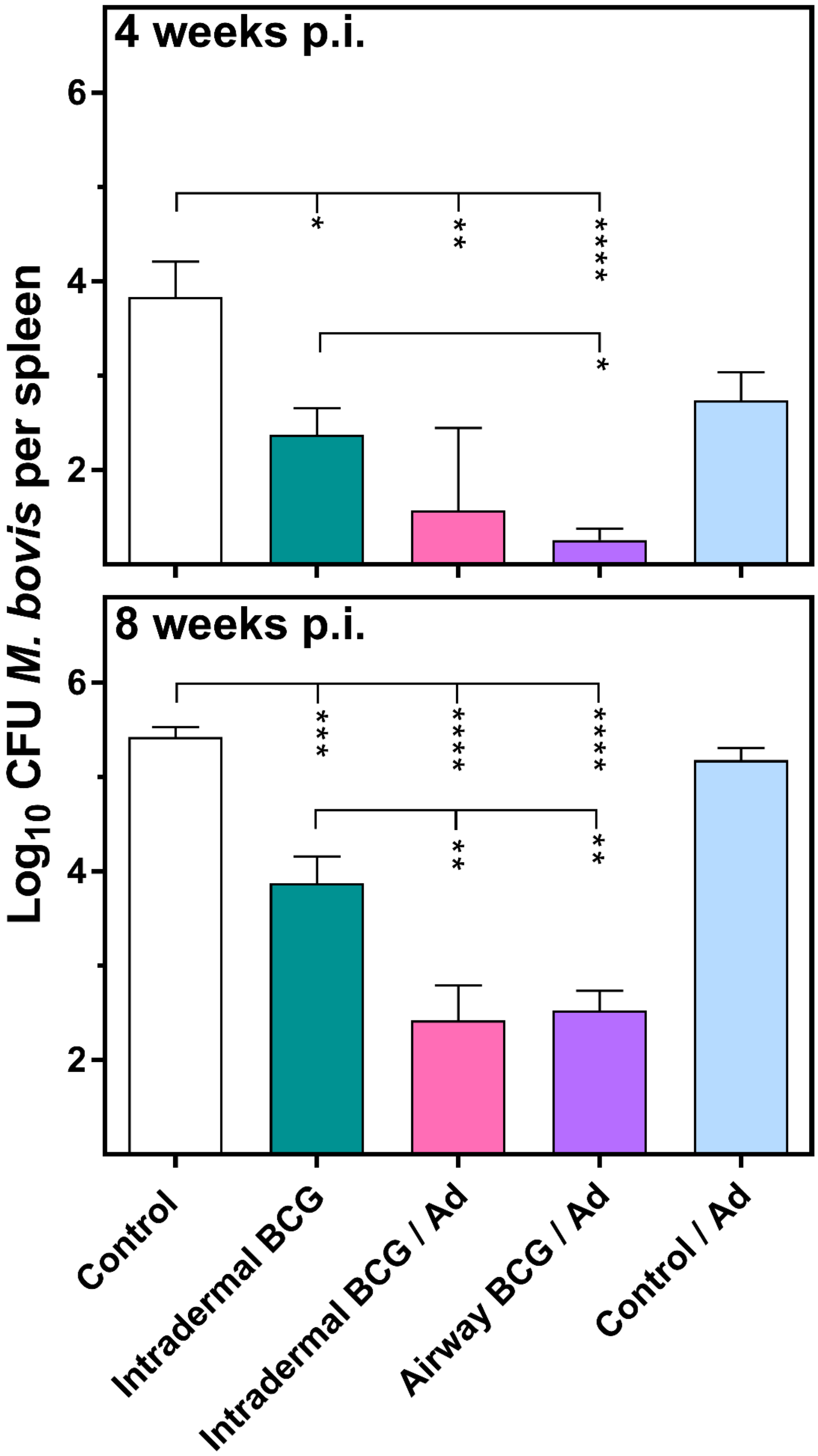

Supplementary Figure S4. Bacterial burden in the spleen following challenge. Groups of mice were immunised and then challenged with ~200 CFU *M. bovis* as per schedule in Fig. 1. Four and eight weeks later the spleens of individual mice in equivalent groups were removed, homogenised and bacteria enumerated. Data represent the mean Log_{10} CFU ±SEM (n = 7-14). * $p < 0.05$, ** $p < 0.01$, *** $p < 0.001$ **** $p < 0.0001$, 1-way ANOVA with Tukey's post-hoc test.
